# Reaction Network and Kinetics Model for Neutral Hydrolysis of Poly(ethylene terephthalate)

**DOI:** 10.1021/acs.iecr.6c00014

**Published:** 2026-02-27

**Authors:** Patrícia Pereira, Peter M. Guirguis, Christian W. Pester, Phillip E. Savage

**Affiliations:** † Department of Chemical Engineering, 215863 The Pennsylvania State University, University Park, Pennsylvania 16802, United States; ‡ Department of Materials Science and Engineering, 5972 University of Delaware, Newark, Delaware 19716, United States

## Abstract

We present a reaction network and kinetic model that describes the hydrolytic depolymerization of poly­(ethylene terephthalate) (PET) in water with neutral pH. The network comprises seven reaction pathways. It includes autocatalysis of PET depolymerization by terephthalic acid (TPA) and the generation of monohydroxyethyl terephthalate, bis­(2-hydroxyethyl) terephthalate, benzoic acid, and decomposition products of TPA and ethylene glycol as byproducts. The model can handle PET hydrolysis in the solid state and in the molten state. Parameter estimation was performed by fitting the model to product concentrations from literature for PET hydrolysis over a wide range of times (15 s to 25 h) and nominal reaction temperatures (170–570 °C). The model fits the entire set of experimental concentrations with a mean absolute error of 0.023 M. It also demonstrated the ability to predict product yields from many published studies. The highest TPA yield predicted by the model is 94%, obtained from hydrolysis at 450 °C (instantaneous heating) after a 20 s batch holding time. TPA yields of 100% were not achievable in the model because of TPA decomposition pathways and equilibrium reactions involving byproducts.

## Graphical abstract

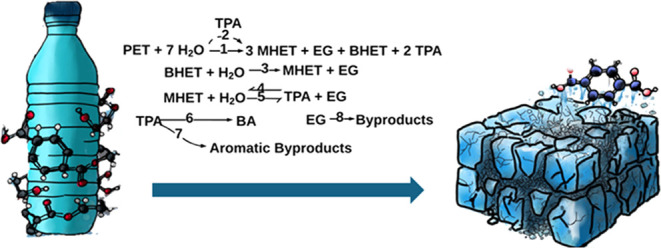

## 1 Introduction

Transforming postconsumer poly­(ethylene terephthalate) (PET) into its original monomers is a strategy to valorize PET waste and reduce the environmental impact of PET production and use. This transformation can be performed by hydrolyzing PET. Water molecules cleave the ester bonds, ideally providing terephthalic acid (TPA) and ethylene glycol (EG) monomers as the ultimate depolymerization products. TPA can be purified and then repolymerized with EG to create virgin PET anew, fostering a circular life cycle for this material. Numerous studies have examined PET hydrolysis under neutral, acidic, and alkaline conditions and over a broad range of temperatures and times.^1–8^

Kinetic models are important for engineering work as they facilitate the design, control, and optimization of chemical processes. For PET hydrolysis, the depolymerization rate is primarily proportional to the concentration of PET, while the concentration of water, being present in excess as the solvent, remains essentially constant throughout the reaction.^1,6,8–15^ Analyses of PET hydrolysis reaction kinetics typically used a pseudohomogeneous model for the reacting system or a modified shrinking core approach that treats the reacting solid particle of PET as a system that becomes smaller and smaller as water molecules attack the external surface^16–24^ and/or the internal surface.^25^

Kinetic models for PET hydrolysis have mainly focused on systems with added heterogeneous or homogeneous catalysts.^8–13,16–24,26^ There has been less work on modeling uncatalyzed hydrolysis, and we briefly review this prior work in the following. Golike and Lasoski^27^ developed a second-order reaction model with the rate limited by diffusion of water within a PET film. Campanelli et al.^28^ modeled neutral hydrolysis of molten (but not solid) PET. Kao et al.^29^ proposed a kinetic model for the hydrolysis of molten PET that included forward and reverse reactions with autocatalysis (half-order with respect to carboxylic acid), a treatment also used in earlier studies.^14,15^ Yang et al.^9^ presented a first-order model for TPA-autocatalyzed hydrolysis of PET. The authors considered only PET conversion, however, and did not include the parallel noncatalytic pathway or the influence of catalyst mass. Čolnik et al.^30^ developed a kinetic model for the evolution of TPA and byproducts (*e.g*., isophthalic and benzoic acids) from neutral PET hydrolysis, but it neglected autocatalysis. However, none of these models were validated against PET hydrolysis data from studies done outside their own laboratories.

Our work is motivated by the fact that none of the extant kinetic models for PET hydrolysis in neutral water include all the aspects that can be important for this system. For example, there is no single model that accounts collectively for (1) PET conversion and TPA formation and decomposition, (2) the formation and subsequent reaction of byproducts such as monohydroxyethyl terephthalate (MHET) and bis­(2-hydroxyethyl) terephthalate (BHET), (3) parallel autocatalyzed and uncatalyzed hydrolysis pathways for PET depolymerization, and (4) hydrolysis of PET in both molten and solid phases. We report herein the development and demonstration of a kinetic model for PET hydrolysis that includes all these features.

## 2 Experimental Methods

We performed hydrothermal reaction experiments with TPA at set point temperatures from 270 to 570 °C and batch holding times ranging from 75 s to 30 min. These experiments provided the required data on the stability of TPA and the extent to which it reacts with EG in hydrothermal systems. Deionized water from an in-house purification system (ion exchange, reverse osmosis, high-capacity ion exchange, UV sterilization, and submicron filtration) served as the reaction medium. Swagelok stainless steel reactors (≈4 mL internal volume), assembled from a stainless-steel port connector and two caps, were loaded with carefully measured amounts of TPA, deionized water, and, when needed, EG, then sealed and placed in a preheated Techne fluidized sand bath (Table S2 details the reactor loadings). Reactions were quenched by removing the reactors from the bath and immediately submerging them in room-temperature water. Once cooled, the reactors were opened, deionized water (10 mL) was added, and the reactor contents were filtered through a Whatman grade-1 cellulose filter paper using a reusable 25 mm filter holder (Cole-Parmer) to isolate solids. Solids were recovered by drying the filters, syringes, and reactors overnight at 80 °C.

To dissolve any TPA in the dried solids, 5 mL of dimethyl sulfoxide (DMSO, Millipore Sigma) was added, and the solution was filtered through a 25 mm PTFE membrane filter (1 μm pore size). The DMSO solutions were analyzed by HPLC using a Waters reversed-phase Symmetry C18 column (5 μm, 150 mm × 4.6 mm) at 40 °C, with detection by a photodiode array detector (SPD-M20A) at 240 nm. The mobile phase comprised HPLC-grade acetonitrile (0.1 mL/min) and a 0.1 v.% formic acid aqueous solution (0.3 mL/min) with a 1 μL injection volume. Calibration curves were generated from analysis of standard solutions of TPA and isophthalic acid (IPA) (both 99% purity, TCI), MHET (Millipore Sigma, 95% purity), BHET (Sigma-Aldrich), and benzoic acid (BA, 99%, Thermo Scientific Chemicals) with known concentrations in DMSO. These calibrations and subsequent HPLC analysis of products provided the moles of each reaction product, with molar yields calculated as the moles of product divided by the moles of PET repeat units initially loaded, as previously reported.^7^ At least three independent runs were performed per condition. Mean values are reported as the best estimates for product yields with standard deviations reflecting run-to-run variability.

## 3 Reaction Network

Figure 1 depicts the reaction network used to describe PET hydrolysis in neutral water. The pathways are based on published studies and preliminary modeling work discussed in subsequent sections.

**Figure 1:**
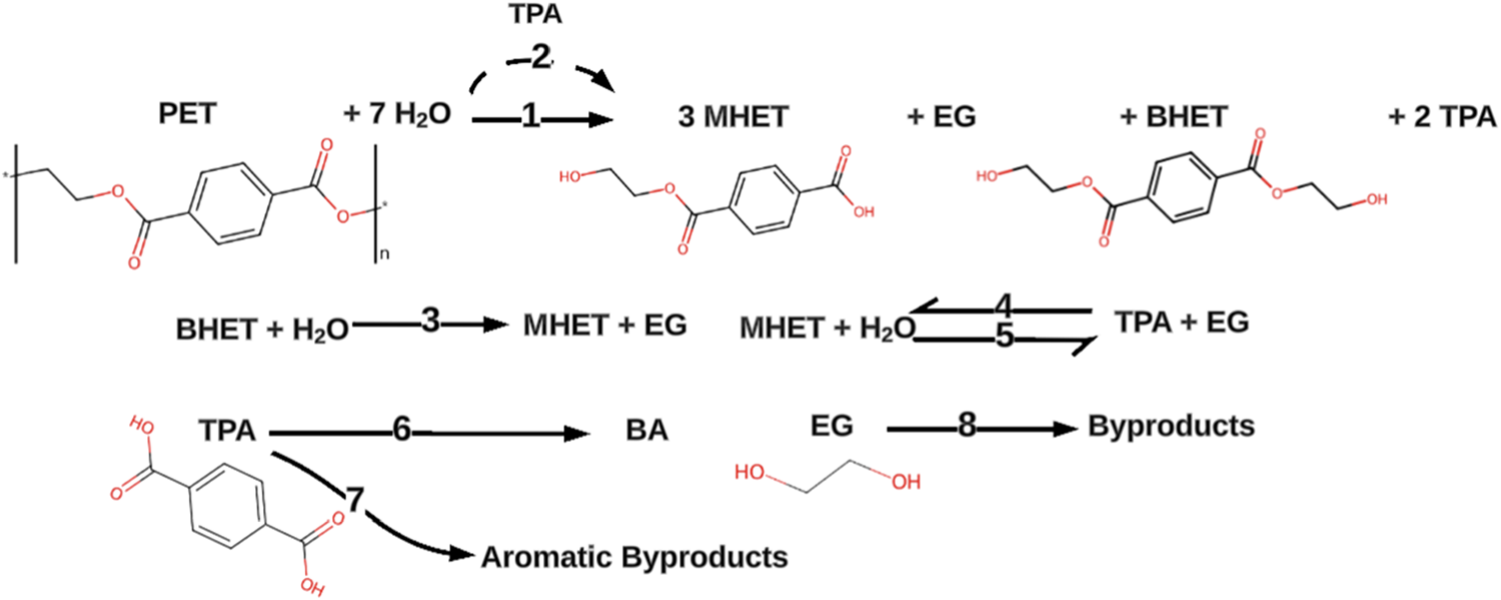
Reaction network for the hydrolysis of PET in neutral water.

The network includes both uncatalyzed and autocatalyzed hydrolytic depolymerization of PET into MHET, EG, BHET, and TPA.^7,31,32^ The stoichiometric coefficients shown for these four products are the statistical values expected if PET hydrolysis proceeds via random scission of the ester bonds in the polymer chain.^33^ The PET reactant is modeled as a set of PET hexamers, as six PET repeat units are needed to produce the statistical distribution of products from hydrolysis in paths 1 and 2.

Hydrolysis of BHET and MHET is included in the network, along with the esterification of TPA with EG.^34^ The reaction network includes pathways to account for the formation of benzoic acid (BA) and other aromatic byproducts.^7,30,35^ These can include isophthalic acid (IPA), mixed esters of TPA with ethylene glycol and diethylene glycol, and other combinations of phthalic acid with ethylene glycol.^36^ EG can also decompose to other products, and its primary decomposition products can further react to form species such as diethylene glycol, triethylene glycol, 1,4-dioxane, and acetaldehyde.^36,37^ We lump together and denominate all these compounds as “Byproducts” in the reaction network.

## 4 Kinetics Model

### 4.1 Model Development

At temperatures above the PET melting point, we treat the reacting system as a single, pseudohomogeneous condensed phase with uniform composition throughout. Species concentrations, including water, are calculated as moles of each divided by the volume of the reaction medium at the reaction temperature.

At temperatures below the PET melting point, the bulk fluid phase is again taken to have uniform composition, but PET is taken to exist as a discrete solid phase into which water molecules can absorb and diffuse.^38–44^ We use correlations to estimate the solubility of water in PET and the diffusion coefficient for water in PET.^38^ The Supporting Information provides details. The volume of the solid PET particle decreases by equal amounts in each direction over time as the PET depolymerizes via interfacial reactions with water. We assume TPA and other products are swept into the pseudohomogeneous bulk fluid phase as they are formed. Figure 2 provides a schematic of the process.

**Figure 2:**
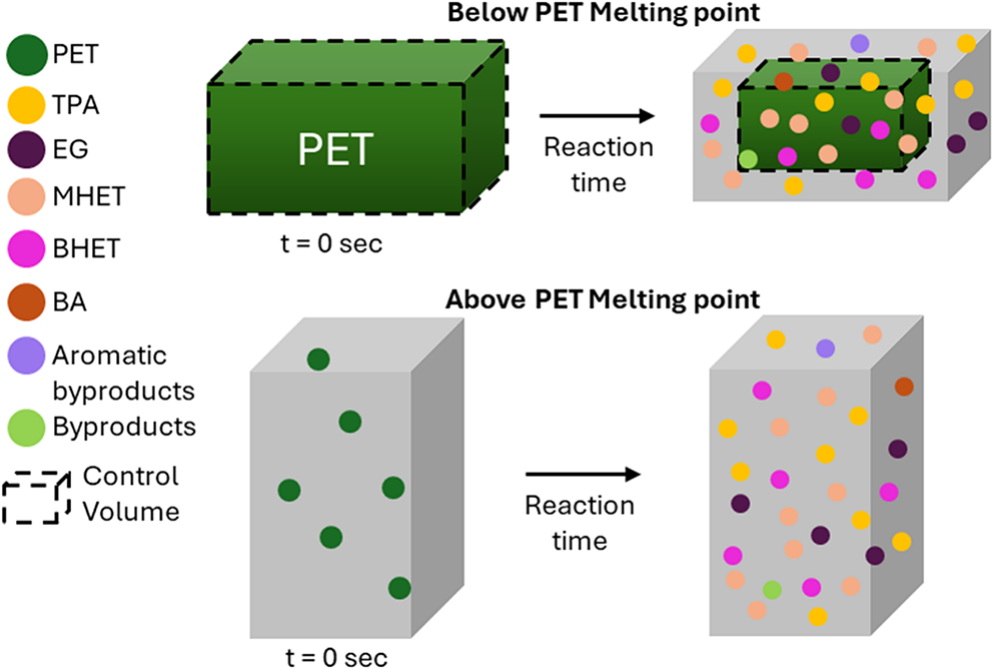
Schematic depiction of PET hydrolysis in the solid and molten states.

We use the average concentrations of water and PET in the solid PET chip to calculate the reaction rates in that phase. The concentration of PET within a solid chip remains fixed until the chip disappears due to depolymerization. The concentration of water within the chip is determined by considering its solubility and diffusivity within the chip and assuming the consumption of water by the hydrolysis reaction is negligible. The Supporting Information provides more details. The temperature at which PET transitions from the solid to the molten state is treated as a fitted parameter. The transition was assumed to be instantaneous. The temperature dependence of the water density was handled using the IAPWS-IF97 Python library.^45^

We use pseudo-first-order kinetics for the effect of the concentration of organic species on reaction rates. The literature provides different approaches for handling the influence of the water concentration on hydrolysis rates in the PET system. Some literature reports PET hydrolysis to be pseudo-first order in water^29,30,33^ while others use a zero-order dependence on the water concentration.^1,8,46^ Rather than assuming a reaction order for water, we use eq 1 to model the effect of the water concentration on the rates of the hydrolysis reactions.

1 $$f\left([H_{2}O]\right)=\frac{k_{s}\left[H_{2}O\right]}{1+k_{s}\left[H_{2}O\right]}$$

The fitted parameter *k*_s_ has units of M^–1^. This equation can admit reaction orders for water that are zero, unity, or any fractional value between.Equations 2–9 show the ordinary differential equations that arise from inserting the rate equations into the design equations for a constant-volume batch reactor. Terms in brackets refer to molar concentrations (M).

2 $$\frac{d\left[\mathrm{PET}\right]}{dt}={(-k}_{1}-k_{2}\left[\mathrm{TPA}\right])\left[\mathrm{PET}\right]f\left([H_{2}O]\right)$$

3 $$\frac{d\left[\mathrm{TPA}\right]}{dt}=\left(2k_{1}\left[\mathrm{PET}\right]+2k_{2}\left[\mathrm{PET}\right]\left[\mathrm{TPA}\right]+k_{5}\left[\mathrm{MHET}\right]\right)f\left([H_{2}O]\right)-k_{4}\left[\mathrm{TPA}\right]\left[\mathrm{EG}\right]-k_{6}\left[\mathrm{TPA}\right]-k_{7}\left[\mathrm{TPA}\right]$$

4 $$\frac{d\left[\mathrm{MHET}\right]}{dt}=\left(3k_{1}\left[\mathrm{PET}\right]+3k_{2}\left[\mathrm{PET}\right]\left[\mathrm{TPA}\right]+k_{3}\left[\mathrm{BHET}\right]-k_{5}\left[\mathrm{MHET}\right]\right)f\left([H_{2}O]\right)+k_{4}\left[\mathrm{TPA}\right]\left[\mathrm{EG}\right]$$

5 $$\frac{d\left[\mathrm{BHET}\right]}{dt}={(k}_{1}\left[\mathrm{PET}\right]+k_{2}\left[\mathrm{PET}\right]\left[\mathrm{TPA}\right]-k_{3}\left[\mathrm{BHET}\right])f\left([H_{2}O]\right)$$

6 $$\frac{d\left[\mathrm{BA}\right]}{dt}=k_{6}\left[\mathrm{TPA}\right]$$

7 $$\frac{d\left[a\mathrm{romatic byproducts}\right]}{dt}=k_{7}\left[\mathrm{TPA}\right]$$

8 $$\frac{d\left[\mathrm{EG}\right]}{dt}={(k}_{1}\left[\mathrm{PET}\right]+k_{2}\left[\mathrm{PET}\right]\left[\mathrm{TPA}\right]+k_{3}\left[\mathrm{BHET}\right]+k_{5}\left[\mathrm{MHET}\right])f\left([H_{2}O]\right)-k_{4}\left[\mathrm{TPA}\right]\left[\mathrm{EG}\right]-k_{8}\left[\mathrm{EG}\right]$$

9 $$\frac{d\left[b\mathrm{yproducts}\right]}{dt}=k_{8}\left[\mathrm{EG}\right]$$

The temperature dependence of the reaction rate constants *k*_i_ was modeled with a modified Arrhenius equation, as

10 $$k_{i}=A_{0,i}T^{n}e^{-E_{a,i}/\mathrm{R T}}$$

where *A*_0,*i*_ is the pre-exponential factor, *E*_a,*i*_ is the activation energy for the *i*th reaction, and *n* is the temperature dependence of the pre-exponential factor, which was treated as a fitted parameter.

## 4.2 Parameter Estimation and Model Discrimination

We used Python to solve the system of ordinary differential equations (ODEs) and to simultaneously perform parameter estimation. When heating times were given in a literature report, we used them in the model and heat-up was assumed to follow a Morse potential.^7^ When no information was given about heating time, we treated the temperature as being constant at the reaction temperature (instantaneous heat up). We used “solve_ivp” from the SciPy (version 1.6.2) library with the “Radau” method as a stiff solver.^47^ Python code for the model is provided at https://github.com/pguirguis/PET_Hydrolysis. We also include an Excel file that calculates product concentrations given the appropriate input data.

Parameter estimation was done by fitting the model to experimental product concentrations from PET hydrolysis in neutral water^1,2,4,7,9,30,37,48–52^ and from new and published studies on reactions of aromatic dicarboxylic acids in water with and without added ethylene glycol. When the literature provided data as product yields or PET conversion, we calculated the corresponding molar concentrations using the reactor loadings and yields or conversions given, along with the appropriate molecular weights. The data are available in a spreadsheet online as part of Supporting Information (See Table S2 and ref 53). We minimized the value of the objective function in eq 11 using the “minimize” function from SciPy using “Nelder–Mead” and “COBYLA” methods in sequence.

11 objectivefunction=∑|Res|

|Res| is the absolute value of the residual (difference between experimental and calculated concentrations) for each species. We used 90% of the experimental data set, selected at random, for parameter estimation. The data set included results from PET hydrolysis,^1,2,7^ new experiments on TPA decarboxylation and esterification, TPA decarboxylation,^53^ and PET hydrolysis with added TPA present initially.^9^ The remaining 10% of the data set was reserved to assess the predictive ability of the model with the fitted kinetic parameters. We also used additional published product yields from studies of PET neutral hydrolysis to assess model predictions.^4,30,37,48–52^ We excluded published TPA yields when these were quantified by a gravimetric method, as this approach can overestimate the yield.^54^ We confined the results used to those from experiments with PET/water mass ratios between 1/3 and 1/25. Table S1 summarizes key aspects of the experimental studies that produced the data used to parametrize the model and test its predictions.

We used the Akaike Information Criterion (AIC) to determine which potential pathways in a reaction network are needed to accurately describe the data. The AIC measures the relative quality of a model with a given data set, and it includes a penalty that increases with the number of parameters in the model. The model with the lowest AIC is generally considered the best because it suggests a good balance between model complexity (number of parameters) and goodness-of-fit. Of the different sets of reaction pathways considered, the reaction network in Figure 1 had the lowest AIC, as reported in Table S3. As an example of pathways explored but omitted from the final model, we considered networks with one or two different classes of “oligomers” between PET and TPA, MHET, BHET, and EG. Including these products and pathways did not improve the ability of the model to fit the data.

Model results indicate the transition from solid-state hydrolysis to hydrolysis in a pseudohomogeneous single fluid phase occurred at 240 °C. This value, within the melting region of PET, is consistent with Liu et al.^55^ reporting that PET did not fully dissolve in hot, compressed water at temperatures up to 240 °C. The optimal value of *n*, the temperature exponent in the modified Arrhenius equation, was found to be 0.65 and *k*_s_ was 0.021 M^–1^, respectively.

Table 1 provides the remaining model parameters, which are the Arrhenius parameters for each rate constant. The activation energies for PET depolymerization (paths 1 and 2) fall within the range reported for PET hydrolysis reactions (90–123 kJ/mol).^1,13^ We used a single activation energy for the hydrolysis of PET, BHET, and MHET, as the AIC score was lower for this approach than for keeping each *E*_a_ as an independent variable. This outcome is reasonable as each reaction involves hydrolytic cleavage of an ester linkage. Esterification of TPA (path 4) exhibits a higher activation energy than the reverse hydrolysis reaction (153 vs 97 kJ/mol, respectively). Additionally, TPA decarboxylation to benzoic acid (path 6) requires a higher activation energy (277 kJ/mol) than the other pathways and has a smaller rate constant, which aligns with benzoic acid being formed less readily than products such as TPA esters.^36,53^

**Table 1:** Arrhenius Parameters for the Rate Constants in the Reaction Network in Figure 1 ^a^

reaction path	log_10_(*A*_0,i_)	*E*_a,i_ (kJ/mol)
1	4.58 ± 0.23	97.3 ± 1.9
2	5.11 ± 0.19	97.3 ± 1.9
3	4.55 ± 0.19	97.3 ± 1.9
4	13.6 ± 0.56	153 ± 7.0
5	9.25 ± 0.40	97.3 ± 1.9
6	13.6 ± 0.44	277 ± 10.
7	1.39 ± 0.10	81.2 ± 1.7
8	4.90 ± 0.43	142.8 ± 5.4

a Units are s, K, L, mol.

## 5 Model Validation

### 5.1 Goodness of Fit

Figure 3 shows how well the model correlates the experimental PET and product concentrations used to determine its parameters. 87% of the product concentrations calculated by the model fall within ±0.05 M of the experimental value and 95% are within ±0.1 M. Most data points for TPA, the molecule of primary interest, reside near the diagonal line, indicating general agreement between calculated and experimental TPA concentrations. The median absolute percentage error for the TPA concentration is 25%. The model is robust enough to fit TPA concentrations from both uncatalyzed and autocatalyzed hydrolytic depolymerization of PET across various experimental conditions.

**Figure 3:**
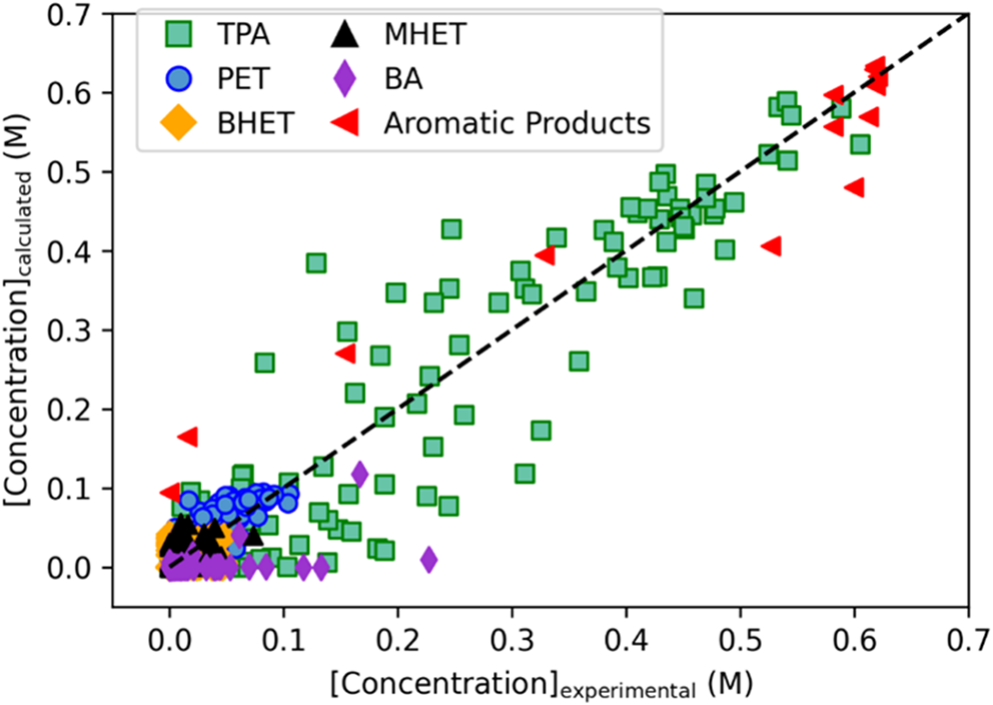
Experimental and calculated concentrations of species for PET neutral hydrolysis.

TPA, BA, and aromatic products are the only products to exceed concentrations of 0.1 M. The model fits these data well. The concentrations of PET, MHET, and BHET are all less than 0.1 M. Note that a given residual for these low-concentration points carries the same statistical weight as the same residual for TPA or one of the products in higher concentrations. Consequently, we expect the average percentage error for these low-concentration points to exceed that for the high-concentration points. Table S3 shows the goodness of fit statistics.

Figure 4 shows representative data comparing the model calculation (lines) and experimental results (discrete points) for TPA yield and PET conversion over time during both isothermal and fast hydrolysis. Fast hydrolysis employs nonisothermal conditions with rapid heating for short batch holding times (<3 min). The model captures well the trends in the experimental data for fast hydrolysis at set-point temperatures of 450 and 540 °C (Panels a and b). For isothermal hydrolysis at 200 °C and 220 °C, the experimental data and model correlation also align closely (panels c and d) for both the TPA yield and PET conversion. The model also handles accurately the influence of catalysis by TPA on the PET conversion. Overall, the model offers an accurate description of the experimental temporal variations of TPA yield and PET conversion for fast and isothermal hydrolysis and for TPA-catalyzed hydrolysis.

**Figure 4:**
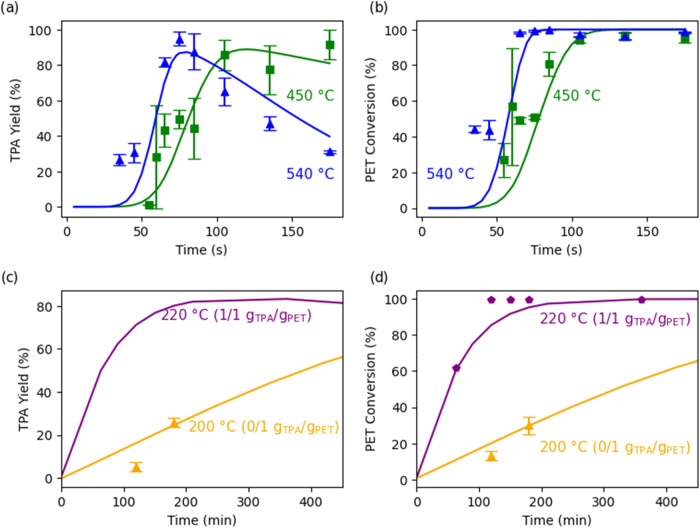
Model (smooth curves) and experimental (discrete points)­[7,9] results for temporal variation of TPA yield and PET conversion for nonisothermal fast hydrolysis (a, b) and isothermal hydrolysis (c, d) at set point temperatures noted. 1/10 w/w PET/water loading.

### 5.2 Predictive Ability

Figure 5 shows how well the model predicts experimental TPA and PET concentrations from PET hydrolysis in neutral water. It shows predictions for data taken from the same studies used to parametrize the model but held in reserve to test predictions, and predictions for data taken from many additional published studies, none of which were used to parametrize the model. The first set of predictions can be viewed as interpolations, while the latter is more akin to extrapolations and represents a more difficult task for the model.

**Figure 5:**
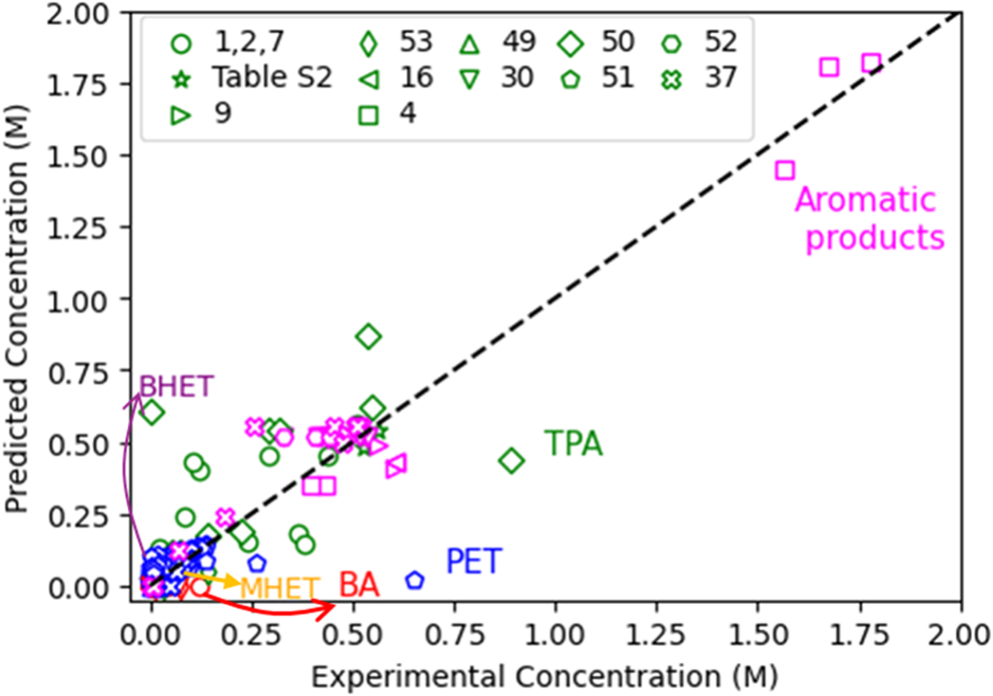
Experimental and predicted concentrations of PET and products from neutral hydrolysis. Numbers in the legend refer to the reference number for those particular data points.

Most data points lie close to the diagonal line, indicating general agreement between predicted and experimental values for the various product concentrations. Indeed, 88% of the predicted concentrations are within 0.1 M of the experimental values. The points furthest from the diagonal line are three for TPA from Subramanya et al.^50^ and one for PET from Goje et al.^51^ These studies used PET/water w/w loadings of 1/5 and 1/1.3, respectively, which are much more PET rich than most other studies. A PET/water w/w loading around 1/10 is more typical. This ratio is known to affect both PET conversion and TPA yield. Table S3 shows that the median error for the predictions was higher than that for fitting (79% vs 46%, respectively), as expected.

The postconsumer PET used in the studies cited in Figure 5 is in the form of thin rectangular chips,^1,2,4,7,30,37,50^ spherical particles^4,9,16,49,51,53^ or cylindrical particles.^50,52^ The studies also included larger cylindrical pellets of virgin PET.^50^ The model generally providing reasonable predictions for TPA yields and PET conversions for these disparate shapes and for both virgin and postconsumer materials indicates it can predict outcomes for hydrolysis of PET in different initial forms.

## 6 Exercising the Model

### 6.1 Exploring the Parameter Space for PET Hydrolysis

Having established that the model can both correlate and predict experimental results for the neutral hydrolysis of PET, we leverage it here to explore the entire time–temperature parameter space. Using the model enables a faster and broader exploration of process variables compared with performing experiments. This exploration can identify conditions that maximize the production of TPA.

The contour plot in Figure 6 illustrates the TPA yields from neutral hydrolysis at various isothermal temperatures and batch holding times when no initial TPA is added to the system. Figure S5 shows the analogous plot for PET conversion. The highest TPA yields occur at temperatures between about 225 and 250 °C for up to 6 h or between 250 and 450 °C for short times. Above 350 °C, the TPA yield rapidly decreases with time after reaching its highest value.

**Figure 6:**
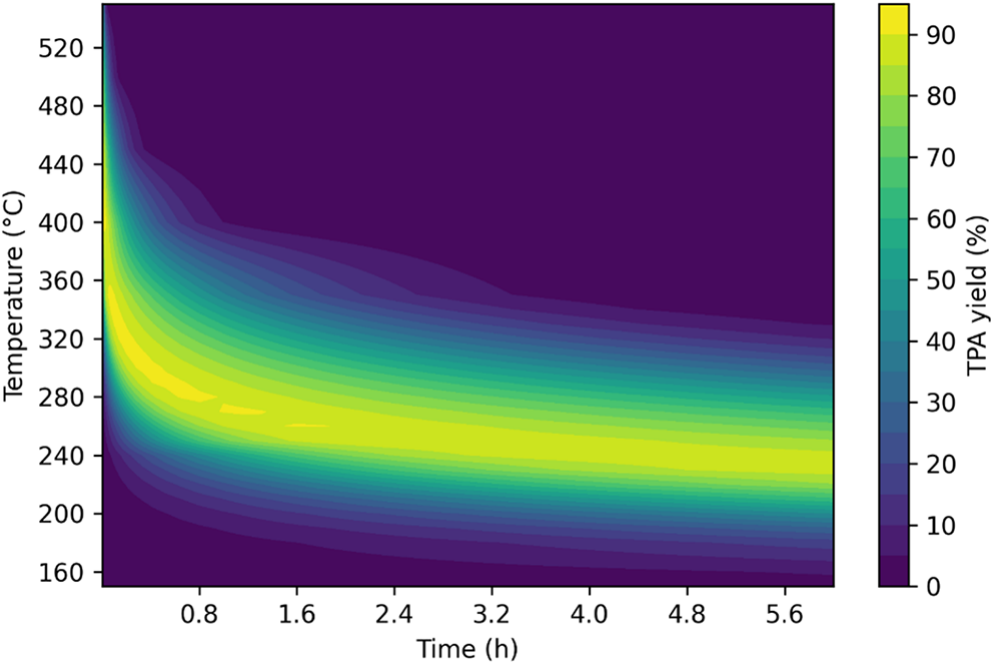
Yield of TPA predicted for isothermal hydrolysis of PET in neutral water, instantaneous heating, no TPA present initially, 1/10 w/w PET to water loading.

We desired to examine the system further and more broadly to find the set of process variables and hydrolysis conditions that maximize the TPA yield. This search considered PET hydrolysis in saturated liquid water up to the critical temperature and in supercritical water at 30 MPa up to 700 °C. Reaction times ranged from 5 s to 55 h. The PET/water ratio was fixed at 1/10 (w/w). We considered initial TPA loadings ranging from 0 to 100% of the initial PET mass and a reactor heat-up that ranged from 1 °C/s to instantaneous. The results of this analysis led to a maximum TPA yield of 94%, obtained from isothermal hydrolysis at 450 °C (instantaneous heating) at a 20 s batch holding time, with no TPA initially added to the reactor. Complete PET decomposition to TPA and EG as the sole products was not possible in the model due to byproduct formation and an equilibrium reaction (paths 4 and 5) involving TPA and MHET. Figure S6 shows how the yields of MHET and BHET are predicted to vary with temperature and time. We note, however, that the predictive ability of the model is poorer for these low-concentration products than it is for TPA, the molecule of primary interest.

### 6.2 Temporal Variation of Product Yields

To get a sense for how the yields of the various products from PET hydrolysis would vary with time, we used the model to predict the yields of all aromatic products from isothermal hydrolysis of PET both below and above its melting point. Figure 7a shows that neutral hydrolysis at 200 °C (in the solid state) requires more than 20 h to completely depolymerize PET, whereas hydrolysis in the molten state at 300 °C (Figure 7a) accomplishes that task in about 10 min. BHET and MHET also hydrolyze to form TPA. The yields of benzoic acid and other byproducts are very low at 300 °C and, hence, not displayed. Hydrolysis at 200 °C, however, is predicted to give a yield of aromatic byproducts that can reach ≈20% at very long reaction times.

**Figure 7:**
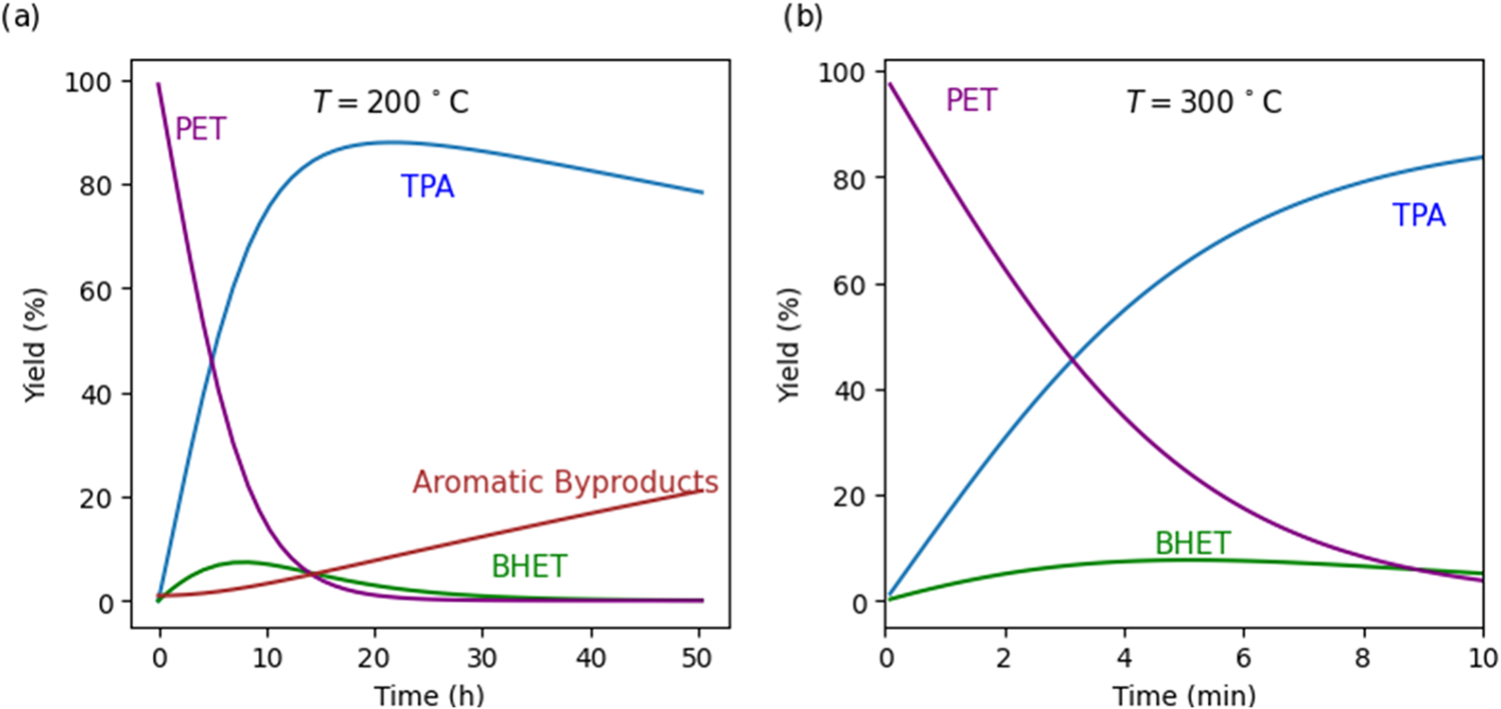
Temporal variation of yields of aromatic products predicted for isothermal hydrolysis of PET in neutral water at (a) 200 °C and (b) 300 °C. instantaneous heating, no TPA present initially, 1/10 w/w PET to water loading.

### 6.3 Change in PET Particle Size

To discover the rate at which solid PET particles shrink during hydrolysis, we used the model to calculate the normalized particle size. Figure 8 shows a PET chip with 0.5 mm thickness persists for about 12 h at 240 °C. The PET particle shrinks faster at higher temperatures, as expected.

**Figure 8:**
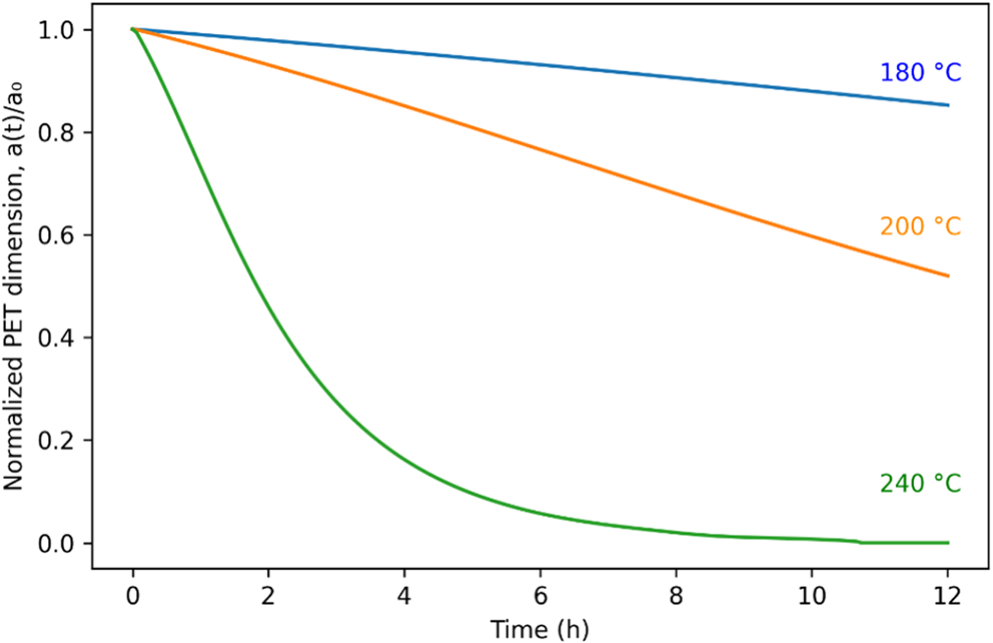
Temporal variation of dimensionless thickness of shrinking rectangular chip of PET (*x* = 5.6 mm, *y* = 8.4 mm, *z* = 0.5 mm).

### 6.4 Reaction Rate Analysis

We calculated the rate for each of the pathways in Figure 1. Figure 9 shows the results for hydrolysis at 200 °C (in the solid state). The variation of the rates with PET conversion and their relative values were about the same for hydrolysis at 300 °C (in the molten state).

**Figure 9:**
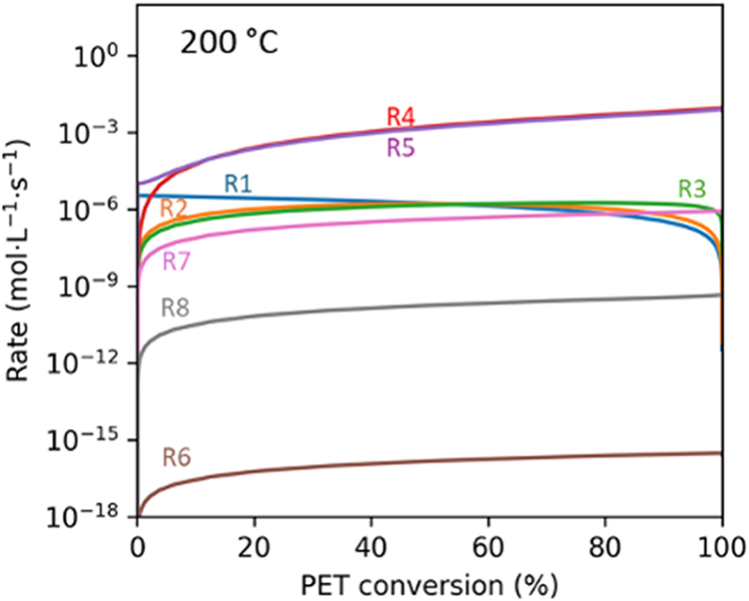
Rate of each reaction path for PET hydrolysis at 200 °C. instantaneous heating, no TPA present initially, 1/10 w/w PET to water loading.

For nearly all PET conversions, pathways 4 and 5 have the fastest rates. These reversible paths connecting MHET and TPA are about 3 orders of magnitude faster than any other pathways. The rapid and nearly equal rates indicate this reaction reaches a quasi-equilibrium state quickly and even at very low PET conversions.

The uncatalyzed reaction (R1) is faster than the autocatalytic hydrolysis reaction (R2), until the PET conversion reaches about 35%. At this point, there is enough TPA in the system for the rates of the parallel paths to be about the same. TPA decomposition to BA (R6) has a rate that is many orders of magnitude slower than any other pathway in the network. The rate of EG decomposition (R8) is also slow, being three to 6 orders of magnitude slower than the main PET hydrolysis reactions.

### 6.5 Sensitivity Analysis

Each rate constant in a complex reaction network can, in principle, influence all the product concentrations. To identify which rate constants were most significant in determining the concentrations of the main hydrolysis products, we performed a sensitivity analysis. We calculated the normalized instantaneous sensitivity coefficients, *S*_ij_ in eq 12, by perturbing each rate constant (*k_j_*) individually by 10% and recording the change in the concentration of each product (Δ*C_i_*), relative to the mean of the original and perturbed concentrations (*C*_*i*,*m*_).

12 $$S_{i,j}=\frac{\frac{{\Delta C}_{i}}{C_{i,m}}}{\frac{{\Delta k}_{j}}{k_{j}}}$$

The larger the sensitivity coefficient, either positive or negative, for product *i*, the more strongly *C*_*i*_ is influenced by the value of the rate constant for path *j*.

Figure 10 shows the sensitivity coefficients for the concentrations of PET and TPA as a function of conversion for isothermal hydrolysis at 220 °C. The SI provides the plots for MHET, BHET, BA, and aromatic byproducts. We show data for the four rate constants to which each species’ concentration was most sensitive.

**Figure 10:**
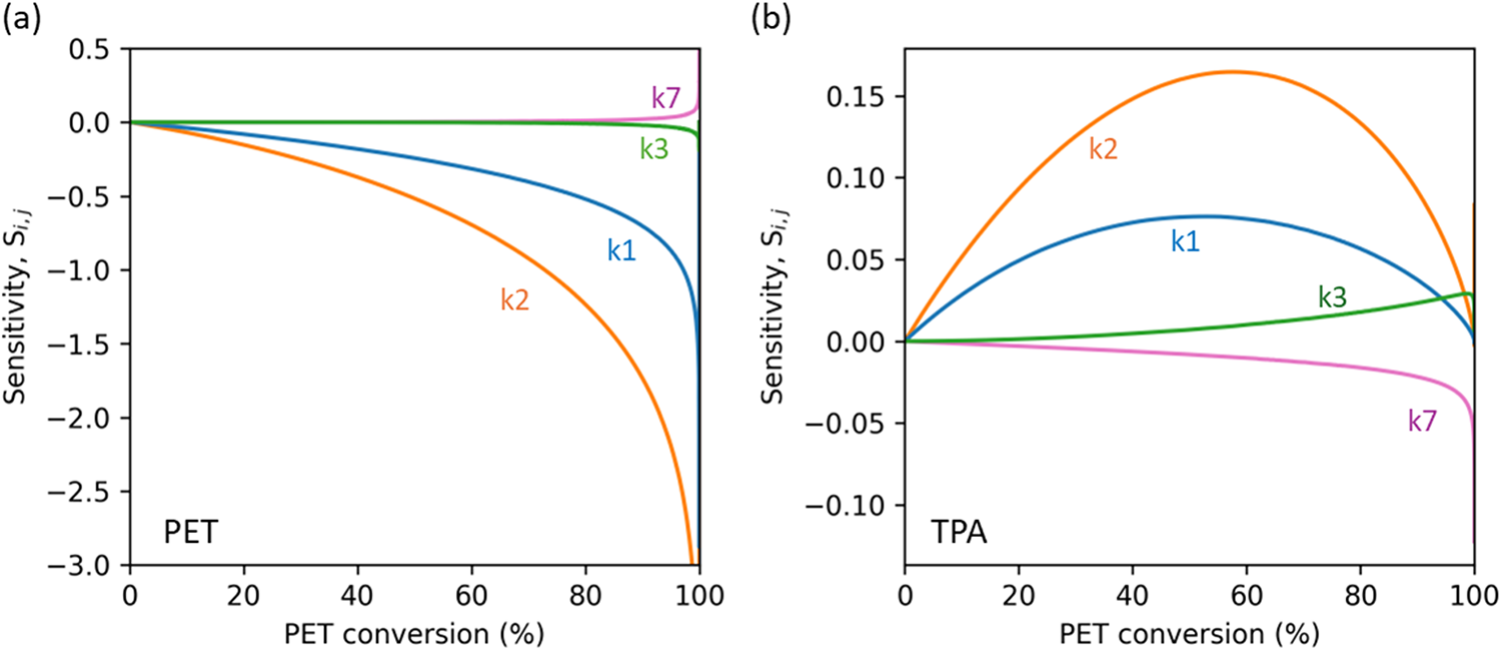
Sensitivity coefficients (*S_ij_*) for PET hydrolysis at 220 °C for (a) PET and (b) TPA. instantaneous heating, no TPA present initially, and 1/10 w/w PET to water loading.

The PET concentration (panel a) is most sensitive to the rate constants for paths 1 and 2, which is expected as these are the main hydrolysis pathways consuming PET. The coefficients are negative because increasing *k*_1_ or *k*_2_ causes a decrease in the PET concentration. The PET concentration shows lesser, but nonzero, sensitivity to the rate constants for paths 3 and 7. Path 3 is the hydrolysis of BHET to make MHET. That product then reacts rapidly in path 5 to generate TPA, which accelerates the rate of the autocatalytic hydrolysis path. Pathway 7 accounts for TPA disappearance, so an increase in this rate constant would decrease the rate of the autocatalytic path and increase the PET concentration.

The TPA concentration (Panel b) is most sensitive to the same four pathways as PET but in the opposite direction. Path 2, autocatalysis, has the most influence, since faster PET hydrolysis produces more TPA. Path 3 shows a positive effect, as increasing the rate of MHET production increases the rate of TPA production in step 5, which follows. Step 7 shows a negative effect on the TPA concentration, as increasing this rate leads to faster consumption of TPA.

The concentration of MHET (Figure S7a) is sensitive to the rate constants for paths 4, 2, and 1 (in that order). These pathways are the irreversible steps that directly produce MHET. The concentration is also sensitive to the rate constant for path 5, which involves MHET consumption. The concentration of BHET (Figure S7b) is most sensitive to the rate constant for path 2 up to about 80% conversion, at which point path 3 has the greatest effect. These steps account for the formation and consumption, respectively, of BHET. The concentrations of BA and the aromatic byproducts (Figure S7c,d) are most strongly influenced by the rate constant for pathway 6 and 7, respectively, as expected. These are the pathways that lead to the production of these compounds.

This discussion has focused on the reaction network at a temperature below the PET melting point. Figure S8 shows similar conclusions can be drawn for hydrolysis above the PET melting point (*e.g*., 300 °C).

## 7 Summary and Conclusions

This work presents a comprehensive kinetic analysis of PET hydrolysis that accounts for multiple reaction pathways, including both uncatalyzed and autocatalyzed PET hydrolysis routes. It incorporates the effects of heating rate, diffusion of water in PET, and byproduct formation. The model can describe the hydrolysis of PET in both molten and solid states. It can accommodate PET particles in any shape and both virgin and postconsumer PET. The model correlates published yields of TPA and other reaction products as well as the conversion of PET under a wide variety of experimental conditions. The agreement between calculated and experimental concentrations demonstrates the robustness and applicability of the model for predicting outcomes in diverse PET neutral hydrolysis scenarios. As such, the model could be useful for conceptual process design, technoeconomic assessments, and dynamic life cycle analyses.

The reversible paths connecting MHET and TPA have the fastest rates in the reaction network. The rapid and nearly equal rates indicate this reaction reaches a quasi-equilibrium state quickly and even at very low PET conversions. This quasi-equilibrium reaction and byproduct formation prevent the achievement of 100% yields of TPA from PET hydrolysis. The calculated concentrations of PET, TPA, and the other primary products are most sensitive to the rate constants for the initial hydrolytic depolymerization paths for PET.

This modeling approach can potentially be used as a framework to quantitatively describe other PET solvolysis methods (*e.g*., glycolysis, acetolysis) as well as catalyzed hydrolysis. A potential limitation in using this approach for other systems is the literature containing insufficient data on product yields over a sufficiently broad range of depolymerization conditions.

## Supplementary Material



## Notes

Derivation for the water concentration inside the PET particle. It also includes calculated water concentration profiles. It lists the studies used to obtain data for parameter estimation and testing predictions, statistics for assessing the performance of the kinetic model, parity plots with concentrations of PET and byproducts for fitted and predicted experimental points, a contour plot for PET conversion as a function of temperature and time, and plots for various sensitivity coefficients (PDF)
