# Supplementary material for: Reaction Network and Kinetics Model for Neutral Hydrolysis of Poly(ethylene terephthalate)
Source: Ind Eng Chem Res. 2026 Feb 27;65(9):4905–14. doi: 10.1021/acs.iecr.6c00014 (PMC12983311; doi:10.1021/acs.iecr.6c00014)
Supplement: Supplementary file 1 [file ie6c00014_si_001.pdf]

**Supporting Information for:**

# Reaction Network and Kinetics Model for Neutral Hydrolysis of Poly(ethylene Terephthalate)

Patrícia Pereira [¶a], Peter M. Guirguis [¶a], Christian W. Pester [b], Phillip E. Savage [a*]

[a] *Department of Chemical Engineering, The Pennsylvania State University, University Park, PA 16802, United States.*

[b] *Department of Materials Science and Engineering, University of Delaware, Newark, DE 19716, United States.*

[¶] These authors contributed equally to this work.

* Corresponding author: P. E. S. (psavage@psu.edu)

## Table of contents

**1. Calculating the water concentration within a solid PET particle** ........ 2

**2. Sources of literature data for parameter estimation and testing model predictions** ........ 8

**3. Statistical performance of the kinetic model** ........ 10

**4. Predicted effect of time and temperature** ........ 11

**5. Sensitivity coefficients for the PET hydrolysis system** ........ 12

## 1. Calculating the water concentration within a solid PET particle

The diffusion of water into and its reaction within the PET particle is governed by **equation 1**, where $[H_2O]$ is the molar concentration of water inside the PET particle, $t$ is time, $D$ is the diffusivity ($cm^2$ $s^{-1}$) of water in PET,[1] $\nabla^2$ is the Laplacian operator, T is temperature in K, and $k_1$ and $k_2$ are the rate constants for paths 1 and 2 in the reaction network in Figure 1 of the article.

$$\frac{\partial[H_2O]}{\partial t} = D\nabla^2[H_2O] - 7k_1[PET]f([H_2O]) - 7k_2[PET]f([H_2O])\,[TPA] \quad (1)$$

$$T > 333\,K \qquad D = 1.523\times10^{-8}e^{-\frac{838.8}{T(t)-233}} \quad (2)$$

$$T \leq 333\,K \qquad D = 31.913e^{-\frac{9068.5}{T(t)}} \quad (3)$$

For the derivation here, we take the control volume (PET chip) to be a rectangular prism with initial dimensions of $x_1$, $y_1$, and $z_1$ measured from the center of mass. The model can also handle cylindrical or spherical PET particles.

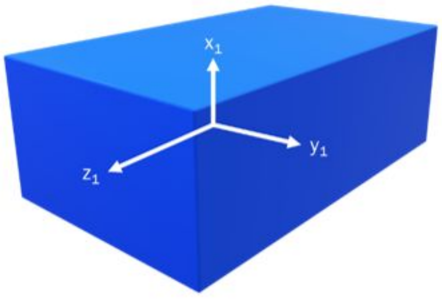


**Figure S1.** PET particle with initial dimensions of $x_1$, $y_1$, and $z_1$ from the center of mass.

The initial condition is $[H_2O]$ = 0 everywhere within the particle at t = 0. Symmetry at the center (*e.g.* x = 0) imposes a zero-flux condition. The boundary conditions are given by the solubility (S, $g_{water}\cdot g_{PET}^{-1}bar^{-1}$) of water in PET, per **Equations 4 and 5** [1] with T in K.

$$T > 333\,K \qquad S = e^{-7.92+\frac{1.59*265}{T(t)-265}} \quad (4)$$

$$T \leq 333\,K \qquad S = e^{-11.6878+\frac{3332}{T(t)}} \quad (5)$$

Neglecting the reaction rate term for the moment to simplify and using the substitution

$$[H_2O] = C_\infty\big(1 - \phi(t,x,y,z)\big) \quad (6)$$

we obtain the full solution for the time-dependent water concentration in the PET particles as

$$[H_2O] = C_\infty \left\{ \begin{matrix} 1 - \Sigma_{n,m,i=1}^{\infty} \mathrm{A_{n,m,i}} \cos\left(\frac{(2n-1)\pi}{2x_1}x\right) cos\left(\frac{(2m-1)\pi}{2y_1}y\right) \\ \cos\left(\frac{(2i-1)\pi}{2z_1}z\right) \mathrm{e}^{-\lambda_{n,m,i}{}^2 D\mathrm{t}} \end{matrix} \right\} \quad (7)$$

with

$$\lambda_{n,m,i}{}^2 = \left(\left(\frac{(2n-1)\pi}{2x_1}\right)^2 + \left(\frac{(2m-1)\pi}{2y_1}\right)^2 + \left(\frac{(2i-1)\pi}{2z_1}\right)^2\right) \quad (8).$$

$C_\infty$ is the molar concentration of water (mol/L) in PET at saturation, and $x_1$, $y_1$, and $z_1$ are the dimensions of the PET particle at any point in time**.** The coefficients $\mathrm{A_{n,m,i}}$ are determined by applying the initial condition.

The average water concentration in the PET control volume, $[H_2O]_{CV}$, can then be obtained by integrating **Equation 7** over the entire particle to obtain **Equation 9**.

$$[H_2O]_{CV} \approx C_\infty \left(1 - \mathrm{e}^{-\left(\left(\frac{\pi}{2x_1(t)}\right)^2 + \left(\frac{\pi}{2y_1(t)}\right)^2 + \left(\frac{\pi}{2z_1(t)}\right)^2\right) D(\mathrm{T(t)})\mathrm{t}}\right) \quad (9).$$

$C_\infty$ can be calculated from Eqn 10

$$C_\infty = \frac{w\,\rho_{wet\,PET}}{(1+w)MW_{water}} \quad (10)$$

Where $w$ is the mass ratio of water to PET at saturation (Eqn 11), $MW_{water}$ is the molecular weight of water, and $\rho_{wet\,PET}$ is the density (g/L) of PET saturated with water, which the literature [1] provides as **equations 12 and 13**.

$$w \; = S\,P^{sat} \quad (11)$$

$$T > 332K \qquad \rho_{wet\,PET} = \; 1556.942 - 0.71614 * T(t) \; (12)$$

$$T \leq 332K \qquad \rho_{wet\,PET} = \; 1418.92 - 0.29935 * T(t) \; (13)$$

**Equation 14** gives the molar concentration of PET (mol/L) inside the particle as a function of temperature. $MW_{PET}$ is the molecular weight of six repeat units of PET, which is the system we use to model the polymer.

$$[PET]_{CV} = \frac{\rho_{wet\,PET}}{(1+w)MW_{PET}} \quad (14)$$

We can now assess whether the reaction rate terms in **equation 1** were safely neglected in the solution process. We first compute the water concentration profile within the shrinking PET particle for the case of diffusion only (no reaction) for an arbitrary reaction duration (2 h) using the lowest mode eigenfunction solution for the corresponding geometry. The total amount of water that would have reacted over that time period, $W_{\text{reacted}} = \int_0^{t_{\text{end}}} (R_{1,1} + R_{2,1})\, dt$, was then calculated and subtracted uniformly from the concentration profile due to diffusion alone.

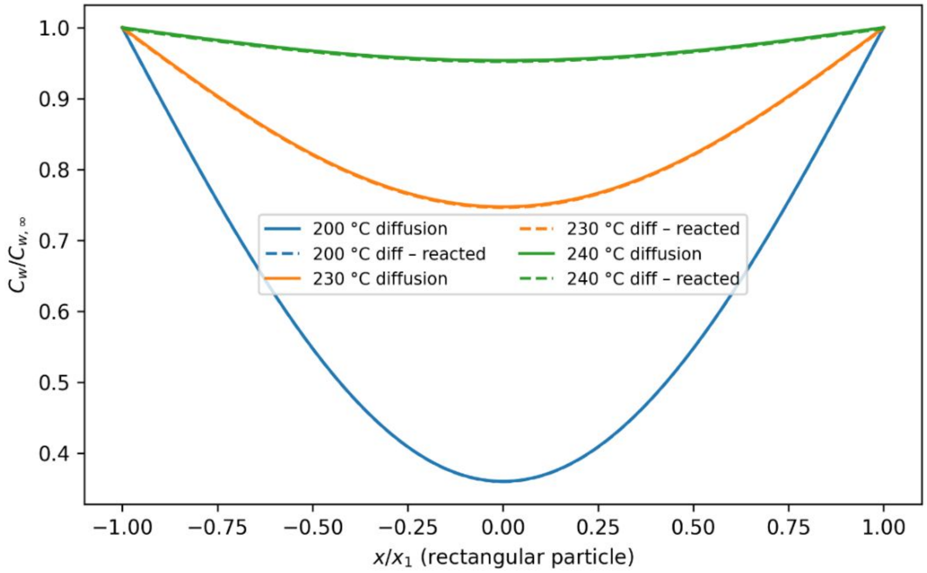


**Figure S2.** Dimensionless water concentration within the PET particle at 2 h considering diffusion alone (solid curves) and the combined effect of diffusion and reaction (dashed curves) on a rectangular chip of initial size *x* = 5.6 mm, *y* = 8.4 mm, *z* = 0.5 mm.

**Figure S2** shows the dimensionless water concentration profiles inside the particle are practically indistinguishable whether considering diffusion alone (solid curves) or diffusion with reaction

(dashed curves) for temperatures ranging from 200 °C to 240 °C. We conclude the reaction terms can be safely neglected in the governing transport equations and, therefore, that the observed rate of PET hydrolysis will be limited by the intrinsic kinetics and not transport of water into and through the PET particle.

This conclusion of negligible diffusion limitations on the rate of hydrolysis of solid PET can be confirmed using the classical models developed for diffusion and reaction in porous heterogeneous catalysts. The governing equations use the Thiele modulus (ϕ), which is given as **Equation 15** for the present reaction system.

$$\phi(t) = L\sqrt{\frac{k_1[PET]}{D}} \quad (15)$$

$L$ is the characteristic length of the PET pellet (e.g., half-thickness for a slab geometry), and [PET] is the instantaneous concentration of PET.

The effectiveness factor (η) in **Equation 16** is a dimensionless measure of how effectively the interior of the PET is being hydrolyzed, relative to the rate at the surface.

$$\eta = \frac{\text{Actual reaction rate}}{\text{Reaction rate with no concentration gradient}} = \frac{tanh\,(\phi)}{\phi} \quad (16)$$

**Figure S3** presents the effectiveness factor as a function of the Thiele modulus. The markers labeled as 100, 200, and 240 °C correspond to the Thiele modulus (evaluated at 2 h) for each temperature for a particle with L = 0.5 mm. The effectiveness factors are all ≈1 indicating minimal internal diffusion resistance under those conditions.

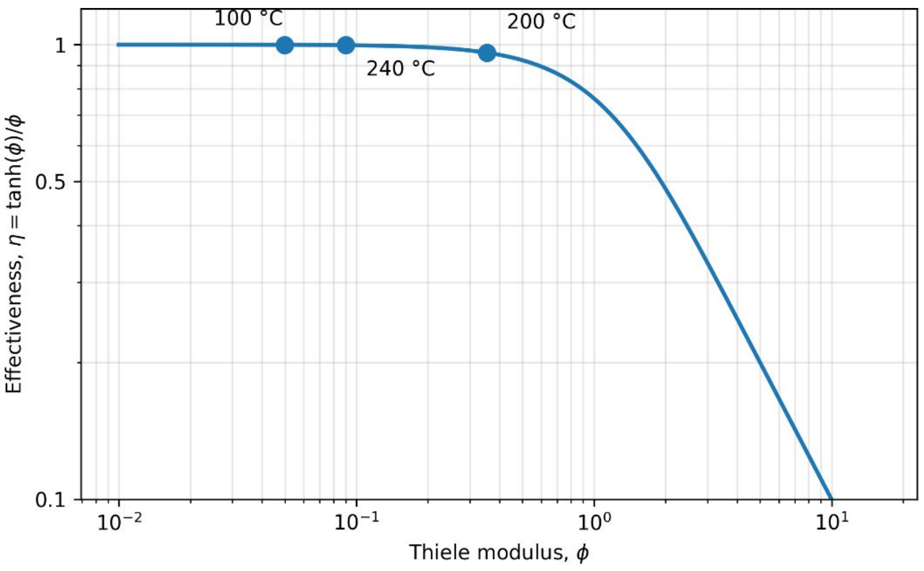


**Figure S3.** Effectiveness factor in relation to Thiele modulus for slab geometry.

**Figure S4** shows a snapshot of the time-dependent concentration for water within a PET particle at 200 °C. After exposure to water for 2 h, the center of the particle is at about 1/3 the water concentration that is present at the surface. At 5 h the water concentration at the center is about ¾ that at the surface and by 10 h the concentration profile is nearly flat.

Above ~200 °C and after several hours, the polymer is essentially saturated with water. We estimate the time required for the concentration of water throughout the particle to reach 99% of the concentration in the surface of the particle is around 6 h for a spherical particle with a characteristic length of 0.5 cm at 200 °C.

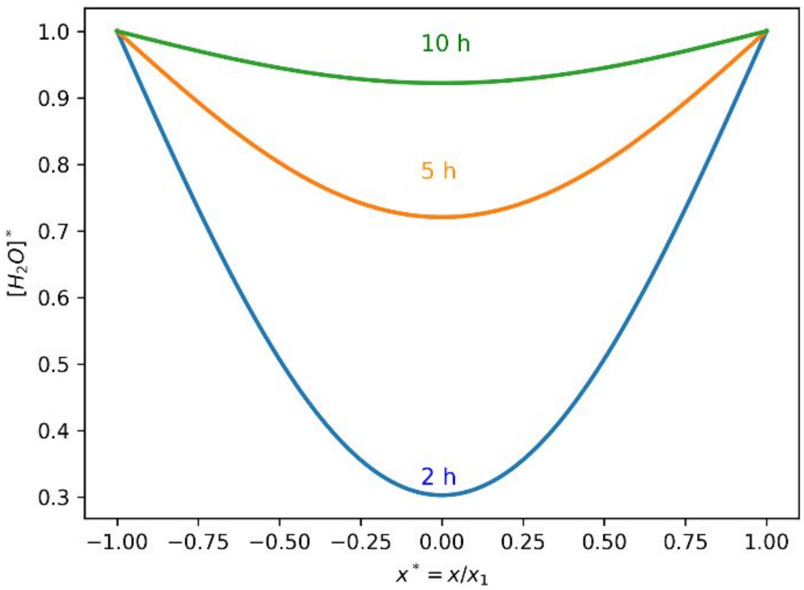


**Figure S4.** Dimensionless water concentration within PET rectangular particle of initial size *x* = 5.6 mm, *y* = 8.4 mm, *z* = 0.5 mm at 200 °C after 2 h, 5 h, and 10 h.

## 2. Sources of literature data for parameter estimation and testing model predictions

**Table S1.** Type of PET in each study and data used in modeling.

| Reference | PET Feedstock | Data Used |
|---|---|---|
| [2–4] | Chips, 5.6 $\pm$ 2.1 mm x 8.4 $\pm$ 2.4 mm, 0.5 mm thick, green bottles, waste | TPA yield and PET conversion |
| [5] | Granules, 60 mesh size, colorless bottles, waste | Yield of aromatic acids* |
| *[6]* | Pellets, 4 x 4 x 4 mm, virgin | PET conversion |
| [7] | Post-consumer flakes, waste | Yield of aromatic acids* |
| [8] | Chips, 3 – 5 mm, water bottles, waste | Yield of aromatic acids* |
| [9] | 150 mesh size waste | PET conversion |
| [10] | Chips, 1 x 1 cm, colorless beverage bottles, waste | Yield of aromatic acids* |
| [11] | Cylindrical pellets, 3.64 ± 0.26 mm × 2.37 ± 0.24 mm, virgin, and single use water bottles | TPA yield |
| [12] | PET waste, 50 – 512.5 mm | PET conversion |
| [13] | Colorless waste bottles, 1/3 mm x 1/3 mm | Yield of aromatic acids* |
| [14] | Isophthalic and Terephthalic acids (not PET) | TPA yield |

**These studies measured gravimetrically the yield of products that precipitated from a basic solution upon acidification. They refer to this material as TPA, but it would include other aromatic acids as well. We took these yields as the sum of all aromatic acids when testing model predictions.*

**Table S2.** Reaction conditions and yields of products from hydrothermal experiments conducted in our laboratory.

| $T_{set\ point}$ (˚ C) | time (sec) | Water loading (mL) | EG loading (mL) | TPA loading (g) | TPA yield (%) | MHET yield (%) | IPA yield (%) |
|---|---|---|---|---|---|---|---|
| 270 | 1800 | 2.92 | 0.098 | 0.292 | $97 \pm 3$ | $2 \pm 1$ | n.d. |
| 308 | 1800 | 2.78 | 0.093 | 0.278 | $91 \pm 0$ | $2 \pm 0$ | n.d. |
| 400 | 175 | 1.43 | 0.048 | 0.143 | $90 \pm 9$ | $2 \pm 1$ | n.d. |
| 500 | 100 | 0.46 | 0.015 | 0.046 | $75 \pm 6$ | $2 \pm 0$ | n.d. |
| 500 | 170 | 0.46 | 0.015 | 0.046 | $72 \pm 5$ | $1 \pm 0$ | n.d. |
| 510 | 75 | 0.44 | 0.015 | 0.044 | $91 \pm 1$ | $5 \pm 1$ | n.d. |
| 510 | 100 | 0.44 | 0.015 | 0.044 | $74 \pm 3$ | $2 \pm 0$ | n.d. |
| 540 | 75 | 0.4 | 0.013 | 0.04 | $93 \pm 8$ | $7 \pm 4$ | n.d. |
| 540 | 170 | 0.4 | 0.013 | 0.04 | $39 \pm 20$ | $1 \pm 0$ | n.d. |
| 570 | 170 | 0.37 | 0.012 | 0.037 | $53 \pm 22$ | $1 \pm 0$ | n.d. |
| 270 | 1800 | 2.92 | 0 | 0.292 | $89 \pm 4$ | $0 \pm 0$ | 0.02 |
| 308 | 1800 | 2.78 | 0 | 0.278 | $100 \pm 0$ | $0 \pm 0$ | < 0.01 |
| 400 | 175 | 1.43 | 0 | 0.143 | $90 \pm 6$ | $0 \pm 0$ | 0.12 |
| 500 | 100 | 0.46 | 0 | 0.046 | $85 \pm 21$ | $0 \pm 0$ | n.d. |
| 500 | 170 | 0.46 | 0 | 0.046 | $72 \pm 13$ | $0 \pm 0$ | n.d. |
| 510 | 75 | 0.44 | 0 | 0.044 | $90 \pm 2$ | $0 \pm 0$ | 0.2 |
| 510 | 170 | 0.44 | 0 | 0.044 | $65 \pm 17$ | $0 \pm 0$ | n.d. |
| 540 | 75 | 0.4 | 0 | 0.04 | $98 \pm 7$ | $0 \pm 0$ | < 0.01 |
| 540 | 170 | 0.4 | 0 | 0.04 | $59 \pm 20$ | $0 \pm 0$ | n.d. |
| 570 | 175 | 0.37 | 0 | 0.037 | 82 | 0 | 0.19 |

## 3. Statistical performance of the kinetic model

**Table S3** summarizes statistics assessing the ability of the kinetic model for PET hydrolysis to fit and predict experimental data. The first row shows that the model fits the 509 experimental concentrations of PET, TPA, byproducts, etc. with negligible bias (mean residual of nearly zero (0.0008 M)) and with a low median absolute error (0.0101 M). The median absolute percentage error (MdAPE) was 46%. This value being large is an expected reflection of the parameter estimation protocol using the absolute, rather than percentage residual as the objective function. The second row shows the errors from the model were larger when making predictions rather than correlating the data, as expected. The subsequent rows provide the same information for each molecule in the reaction network.

**Table S3.** Summary of statistics assessing performance of the kinetic model

| | | Residuals (M) | | | | |
|---|---|---|---|---|---|---|
| **Data Set** | **# points** | **Mean** | **Mean Abs** | **Median Abs** | **MdAPE (%)** | **AIC** |
| All - fitting | 509 | 0.0008 | 0.0228 | 0.0101 | 46 | -3199 |
| All - predicting | 232 | -0.0118 | 0.0581 | 0.0359 | 79 | (-) |
| Fit - PET | 98 | -0.0093 | 0.0131 | 0.0096 | 57 | |
| Fit - TPA | 107 | 0.0067 | 0.0550 | 0.0458 | 25 | |
| Fit - MHET | 100 | -0.0049 | 0.0123 | 0.0071 | 86 | |
| Fit - BHET | 100 | 0.0007 | 0.0122 | 0.0092 | 43 | |
| Fit - BA | 91 | 0.0130 | 0.0132 | 0.0022 | 95 | |
| Predict - PET | 108 | 0.0005 | 0.0483 | 0.0338 | 100 | |
| Predict - TPA | 26 | -0.0572 | 0.1504 | 0.0884 | 58 | |
| Predict - MHET | 12 | -0.0031 | 0.0159 | 0.0096 | 82 | |
| Predict - BHET | 12 | -0.0020 | 0.0077 | 0.0017 | 1 | |
| Predict - BA | 24 | 0.0199 | 0.0199 | 0.0038 | 100 | |

## 4. Predicted effect of time and temperature

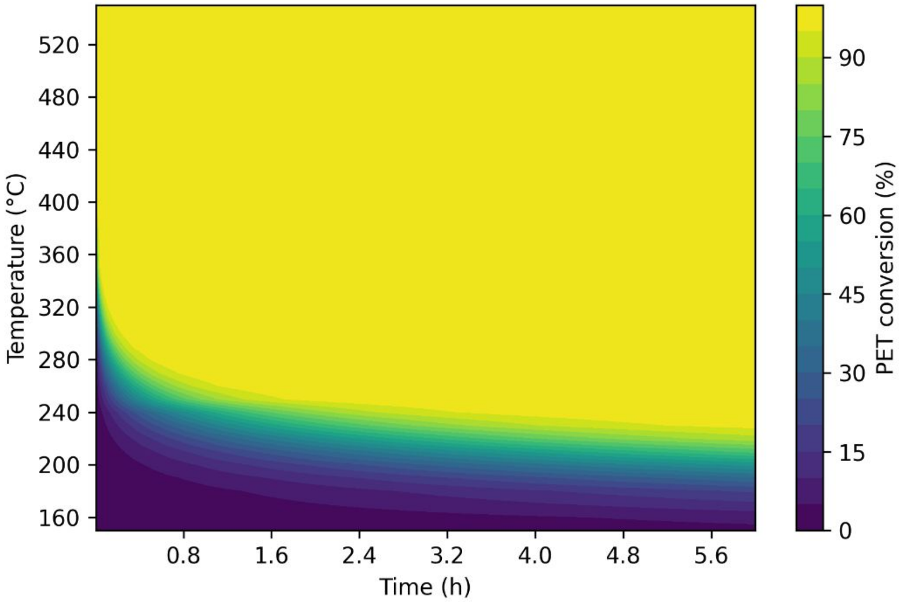


**Figure S5.** Heat map showing conversion of PET from isothermal hydrolysis in neutral water, instantaneous heating to the reaction temperature, no TPA present initially, 1/10 w/w PET to water loading.

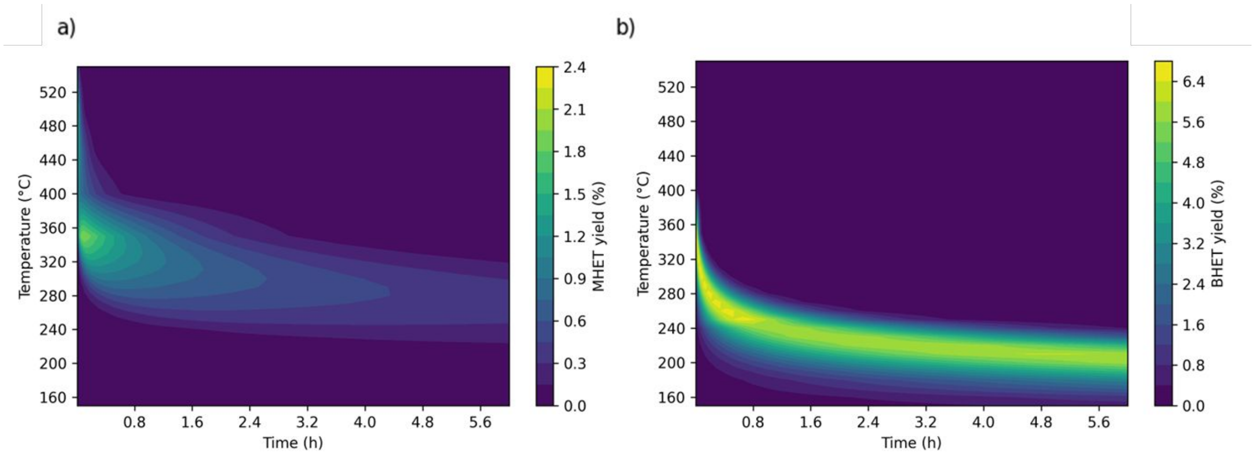

**Figure S6.** Yields of a) MHET, and b) BHET predicted for isothermal hydrolysis of PET in neutral water, instantaneous heating, no TPA present initially, 1/10 w/w PET to water loading.

## 5. Sensitivity coefficients for the PET hydrolysis system

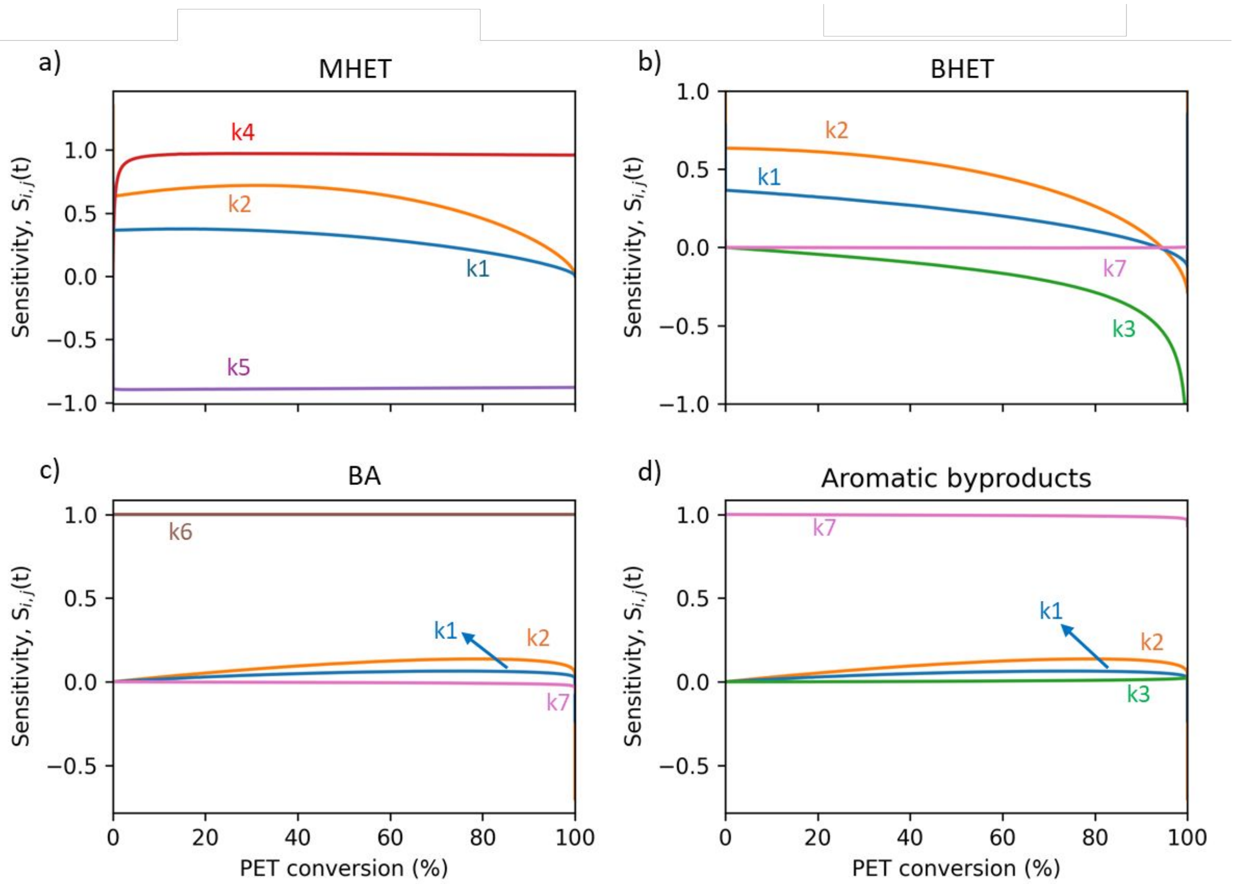


**Figure S7.** Sensitivity coefficients ($S_{ij}$) for PET hydrolysis at 220 °C for a) MHET, b) BHET, c) BA, and d) aromatic byproducts. Instantaneous heating to the reaction temperature, no TPA present initially, and 1/10 w/w PET to water loading.

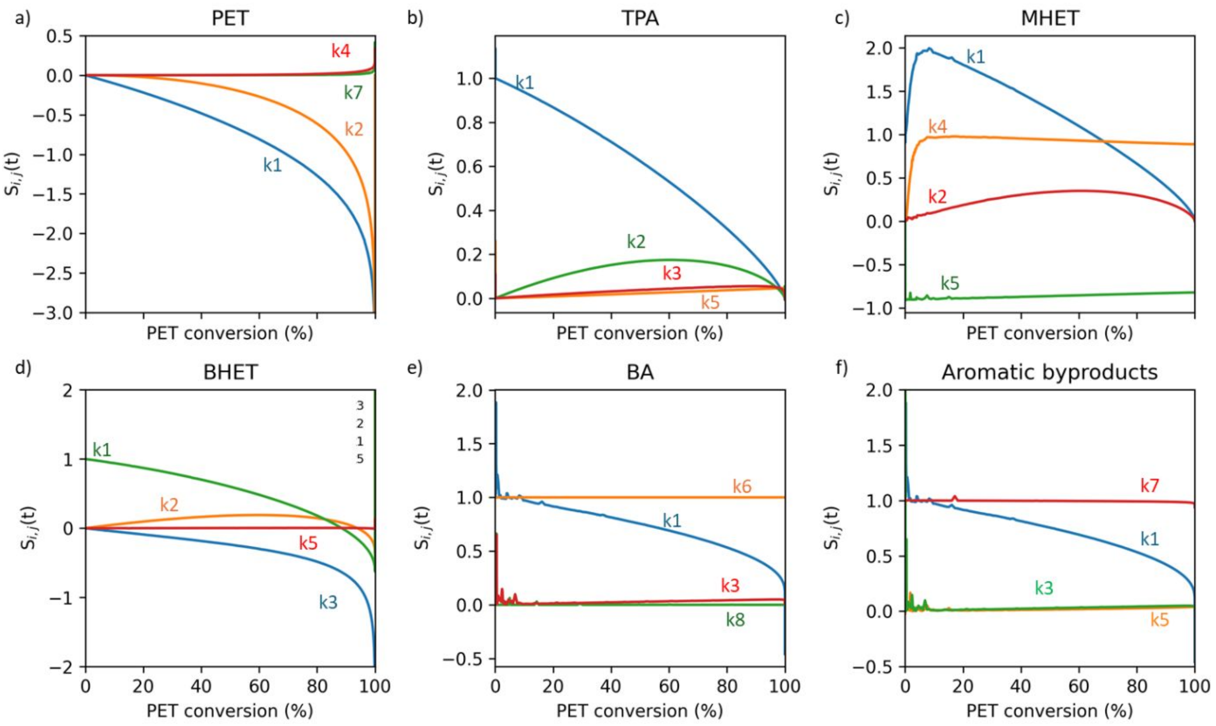


**Figure S8.** Sensitivity coefficients ($S_{ij}$) for PET hydrolysis at 300 °C for a) PET, b) TPA, c) MHET, d) BHET, e) BA, and f) aromatic byproducts. Instantaneous heating to the reaction temperature, no TPA present initially, and 1/10 w/w PET to water loading.

## References


[1] H. Eslami, F. Müller-Plathe, Water permeability of poly(ethylene terephthalate): A grand canonical ensemble molecular dynamics simulation study, J Chem Phys 131 (2009). https://doi.org/10.1063/1.3274805.

[2] P. Pereira, C.W. Pester, P.E. Savage, Neutral hydrolysis of post-consumer polyethylene terephthalate waste in different phases, ACS Sustain Chem Eng 11 (2023) 7203–7209. https://doi.org/10.1021/acssuschemeng.3c00946.

[3] P. Pereira, P.E. Savage, C.W. Pester, Acid catalyst screening for hydrolysis of post-consumer PET waste and exploration of acidolysis, Green Chemistry 26 (2024) 1964–1974. https://doi.org/https://doi.org/10.1039/D3GC03906D.

[4] P. Pereira, W. Slear, A. Testa, K. Reasons, P. Guirguis, P.E. Savage, C.W. Pester, Fast hydrolysis for chemical recycling of polyethylene terephthalate (PET), RSC Sustainability 2 (2024) 1508–1514. https://doi.org/10.1039/d4su00034j.

[5] W. Yang, R. Liu, C. Li, Y. Song, C. Hu, Hydrolysis of waste polyethylene terephthalate catalyzed by easily recyclable terephthalic acid, Waste Management 135 (2021) 267–274. https://doi.org/10.1016/J.WASMAN.2021.09.009.

[6] O. Sato, K. Arai, M. Shirai, Hydrolysis of poly(ethylene terephthalate) and poly(ethylene 2,6-naphthalene dicarboxylate) using water at high temperature: Effect of proton on low ethylene glycol yield, Catal Today 111 (2006) 297–301. https://doi.org/https://doi.org/10.1016/j.cattod.2005.10.040.

[7] S.D. Mancini, M. Zanin, Optimization of Neutral Hydrolysis Reaction of Post-consumer PET for Chemical Recycling, Progress in Rubber, Plastics and Recycling Technology 20 (2004) 117–132. https://doi.org/10.1177/147776060402000202.

[8] C.N. Onwucha, C.O. Ehi-Eromosele, S.O. Ajayi, M. Schaefer, S. Indris, H. Ehrenberg, Uncatalyzed Neutral Hydrolysis of Waste PET Bottles into Pure Terephthalic Acid, Ind Eng Chem Res 62 (2023) 6378–6385. https://doi.org/10.1021/acs.iecr.2c04117.

[9] V.S. Zope, S. Mishra, Kinetics of neutral hydrolytic depolymerization of PET (Polyethylene terephthalate) waste at higher temperature and autogenious pressures, J Appl Polym Sci 110 (2008) 2179–2183. https://doi.org/10.1002/APP.28190.

[10] M. Čolnik, D. Pečar, Ž. Knez, A. Goršek, M. Škerget, Kinetics Study of Hydrothermal Degradation of PET Waste into Useful Products, Processes 2022, Vol. 10, Page 24 10 (2021) 24. https://doi.org/10.3390/PR10010024.

[11] S.M. Subramanya, Y. Mu, P.E. Savage, Effect of Cellulose and Polypropylene on Hydrolysis of Polyethylene Terephthalate for Chemical Recycling, ACS Engineering Au 2 (2022) 507–514. https://doi.org/10.1021/ACSENGINEERINGAU.2C00024.

[12] A.S. Goje, S.A. Thakur, V.R. Diware, S.A. Patil, P.S. Dalwale, S. Mishra, Hydrolytic depolymerization of poly(ethylene terephthalate) waste at high temperature under autogenous pressure, Polymer -

Plastics Technology and Engineering 43 (2004) 1093–1113. https://doi.org/10.1081/PPT-200030031.

[13] A. Cəta, M. Micləu, I. Ienaşcu, D. Ursu, C. Tənasie, M.N. Ştefənuţa, Chemical recycling of Polyethylene Terephthalate (PET) waste using Sub- and supercritical water, Revue Roumaine de Chimie 60 (2015) 579–585.

[14] J.B. Dunn, M.L. Burns, S.E. Hunter, P.E. Savage, Hydrothermal stability of aromatic carboxylic acids, J Supercrit Fluids 27 (2003) 263–274. https://doi.org/10.1016/S0896-8446(02)00241-3.
